# Monitoring nitrate reduction: hydrogeochemistry and clogging potential in raw water wells

**DOI:** 10.1007/s10661-021-08880-y

**Published:** 2021-02-05

**Authors:** F. Ortmeyer, K. Volkova, F. Wisotzky, S. Wohnlich, A. Banning

**Affiliations:** Hydrogeology Department, Ruhr-Universität Bochum, Universitätsstraße 150, 44801 Bochum, Germany

**Keywords:** Hydrochemistry, Nitrate degradation, Well clogging, Admixture, Germany

## Abstract

The mainly agricultural input of NO_3_^-^ and compliance with drinking water guideline values pose major challenges for many water suppliers. Additionally, associated changes in hydrochemistry, especially concerning products of NO_3_^-^ reduction (Fe^2+/3+^, Mn^2+/4+^, Ca^2+^, Mg^2+^, SO_4_^2-^, HCO_3_^-^) and subsequent reactions, can have a major influence on mineral saturation states and well yield: well productivity can be strongly reduced by mineral precipitation and silting. To evaluate hydrogeochemical evolution and clogging potential for a given well field, thorough hydrochemical and geochemical investigations are required. Therefore, time-dependent and depth-specific ion concentrations in water samples (*n* = 818) were analysed in a catchment area of a waterworks in western Germany. The sediments of the aquifers were extensively investigated for their geochemistry (CS, scanning electron microscope, aqua regia digestion and dithionite solution; *n* = 253). In addition, PhreeqC was used to model saturation indices in order to identify possible mineral precipitation in the wells. Results show a high NO_3_^-^ input into deep wells screened in Tertiary sediments due to an admixture of Quaternary groundwater. Directly at the Quaternary-Tertiary boundary, chemolithotrophic NO_3_^-^ reduction consuming pyrite occurs. Protons released during the process are pH-buffered by dissolving carbonate minerals. Overall, the hydrochemistry and especially the saturation indices are strongly influenced by NO_3_^-^ reduction and its degradation products. A change in well yield has not yet been observed, but future clogging by ochre formation or sintering cannot be excluded.

## Introduction

Nitrogen is an essential plant nutrient and important for increasing agricultural productivity. However, if nitrogen in the form of NO_3_^-^ enters groundwater, it can have effects on ecosystems and human health (Rivett et al. 2008). Therefore, the threshold value for NO_3_^-^ of the European Drinking Water Directive (98/83/EC 1998) is 50 mg/l, to achieve a good groundwater quality. Additionally, the role of hydrological processes and ecological functioning in groundwater are important in this context, which is described by the Water Framework Directive of the European Community (EC 2000). Caschetto et al. (2014) describe the relationship between groundwater and surface water, which has a considerable impact on the distribution of nitrogen pollution and on the invertebrate riverine biota: in the river floodplain, the nitrogen cycle was governed by complex groundwater flow and anthropogenic impact. Despite rather low nitrogen concentrations in groundwater and surface water, considerable ammonium concentrations were observed due to the complex redox dynamics of the setting (Caschetto et al. 2014). This still incompletely understood link between groundwater alteration and ecological response will have to be addressed in future research. It is assumed that NO_3_^-^ pollution will continue to increase in the future as NO_3_^-^ degradation capacity in aquifers decreases and rising concentrations are additionally expected due to climate change (Schwientek et al. 2017; Ortmeyer et al. 2020). Nevertheless, NO_3_^-^ can still be degraded in the aquifer (Rivett et al. 2008) and influence the entire hydrochemistry through denitrification.

Nitrate reduction can essentially be divided into two processes. In chemoorganotrophic NO_3_^-^ reduction, also known as heterotrophic denitrification, NO_3_^-^ is reduced by organic carbon (C_org_). Reaction products are molecular N_2_, bicarbonate, carbon dioxide and water (Eq. 1). This has already been investigated by Trudell et al. (1986), Starr and Gilham (1989) and Smith et al. (1991), amongst others.

1 $$ 5\ {\mathrm{CH}}_2\mathrm{O}+4\ {\mathrm{N}\mathrm{O}}_{3^{-}}\kern0.5em \to \kern0.5em 2\ {\mathrm{N}}_2+4\ {\mathrm{H}\mathrm{CO}}_{3^{-}}+{\mathrm{CO}}_2+3\ {\mathrm{H}}_2\mathrm{O} $$

The chemolithotrophic NO_3_^-^ reduction is also called autotrophic denitrification. In this process, NO_3_^-^ is converted into molecular nitrogen (Eq. 2) by reduced sulphur compounds such as pyrite (FeS_2_). Additionally, SO_4_^2-^, Fe^2+^ and water are formed. This reaction has also been extensively investigated by Kölle et al. (1982), Jorgensen et al. (2009) and Zhang et al. (2012), for example.

2 $$ 5\ \mathrm{Fe}{\mathrm{S}}_2+14\ {\mathrm{N}\mathrm{O}}_{3^{-}}+4\ {\mathrm{H}}^{+}\kern0.5em \to \kern0.75em 7\ {\mathrm{N}}_2+10\ {{\mathrm{S}\mathrm{O}}_4}^{2-}+5\ {\mathrm{Fe}}^{2+}+2\ {\mathrm{H}}_2\mathrm{O} $$

Rarely, NO_3_^-^ can also be reduced by the input of methane (Bhattacharjee et al. 2016). However, this reaction is not observed in the present investigation.

The input of NO_3_^-^ and the associated NO_3_^-^ reduction can strongly influence the hydrogeochemical composition of groundwater. Thus, solids might precipitate due to the release of reaction products of NO_3_^-^ reduction altering mineral saturation equilibria.

The specific well yield describes the efficiency of a well. Wells are often exposed to ageing processes, so the yield decreases with increasing running time and production. This leads to clogging of the filter sand and gravel as well as the filter tubes (Houben 2004; Houben and Treskatis 2012).

The most frequent cause of well ageing in Germany is incrustation, which accounts for almost 80% of all well ageing. Incrustations are subdivided into ochre formation and sintering. A total of 68% of well ageing is caused by ochcification, which leads to precipitation of Fe and Mn oxides and hydroxides (Grossmann 2000). These precipitates occur in the neutral pH range when oxidising agents such as O_2_ are present (Tamura et al. 1976). Iron precipitates account for a much larger proportion than Mn precipitates (Houben 2003).

The precipitation of carbonate minerals is called sintering. This occurs when the Ca carbonate equilibrium changes. If the flow velocity is considerably accelerated, the carbonic acid is stripped from the solution. This process takes place, e.g. during the entry of groundwater into a well chamber, or when the water level drops sharply. If carbonic acid is stripped, carbonate minerals such as calcite (CaCO_3_) and dolomite (CaMg(CO_3_)_2_) can precipitate from the solution. In reduced horizons, ankerite (CaFe(CO_3_)_2_) and siderite (FeCO_3_) may additionally precipitate (Langguth and Voigt 2004). A mixture of groundwaters of different compositions can also lead to sintering under special hydrogeochemical conditions (Rinder et al. 2013).

An incorrect dimensioning of the filter openings or the grain size of the filter sand or gravel can lead to well sanding. In this case, sand from the aquifer enters the well. Hydraulic overloading, excessive lowering of the groundwater level and inadequate well development can also lead to silting up (Langguth and Voigt 2004). With a share of 14% of well ageing, well silting accounts for only a relatively small proportion (Grossmann 2000).

Corrosion and biofilms play a rather subordinate role with regard to well yield. The most common form of corrosion is the formation of rust. One reason for this may be the incorrect choice of lining materials with regard to groundwater properties. Chemical regeneration of wells by the use of certain chemicals can also cause corrosion (Langguth and Voigt 2004). Microbial deposits often occur in nutrient-rich waters. Organic carbon, nitrogen and phosphorus, which are decisive for biological growth in groundwater, are often found especially in bank filtrate. Hijn and Van der Kooij (1992) point out that even small concentrations of 0.01 mg/l organic carbon can cause a biological reduction in aquifer permeability.

In this study, an aquifer consisting of hydraulically linked Tertiary and Quaternary sediments is investigated in which a flow of Quaternary water into the underlying Tertiary sediments occurs due to groundwater extraction from the latter. Therefore, special attention is paid to the effects of an admixture of oxidising and nitrate-rich shallow groundwater on the extracted primarily reducing deeper raw water. This admixture strongly changes the hydrochemistry by NO_3_^-^ reduction and its subsequent reactions, potentially leading to decreasing raw water quality and mineral precipitation with associated well yield. For this purpose, long-term groundwater hydrochemistry monitoring data were evaluated and aquifer sediments were extensively investigated to determine the effects of NO_3_^-^ reduction and to detect possible precipitation by modelling mineral saturation states.

## Materials and methods

### Study area

The study area is located in the federal state of North Rhine-Westphalia, western Germany, near the river Rhine and the city of Krefeld. The area is morphologically flat, with general groundwater flow directed from south to north. Geologically, it is situated in an intraplate tectonic rift structure, the Lower Rhine Embayment. Due to erosion of the surrounding Variscan Rhenish Massif, the Lower Rhine Embayment was filled with Tertiary marine sediments, mainly fine sands. These are overlain by Quaternary sediments consisting of fluvial coarse sands and gravel (deposition terraces of the river Rhine). Locally, the Quaternary deposits have a thickness of about 37 m and are hydraulically connected to the underlying Tertiary sands.

The waterworks operating in this study area pump about 2 million m^3^/a groundwater for drinking water production. Four shallow wells are screened at a depth of about 40 m b.g.l. within the Quaternary gravels, while 4 deeper wells are screened in the Tertiary fine sands at a depth of about 160 m b.g.l. The size of the waterworks catchment area is about 15 km^2^.

Due to strong agricultural activity in the region, the Quaternary aquifer is strongly influenced by anthropogenic factors. Fertilisation on the fields leads to a nutrient (mainly nitrogen) entry into the shallow aquifer, resulting in widespread exceedance of the NO_3_^-^ threshold value of 50 mg/l of the German drinking water regulation (Trinkw 2001) in groundwater.

### Sampling

In order to investigate and characterise the development of the hydrogeochemical situation, a comprehensive sampling and analysis programme was carried out.

For this purpose, sediments were sampled and extensively characterised from four boreholes. Drilling and sampling for all boreholes took place in June and July 2018, i.e. in a generally warm and dry summer. Drilling was carried out in the Quaternary aquifer (the upper ca. 40 m b.g.l.) using the dry drilling method (*n* = 8 at each borehole). In the Tertiary aquifer, at one borehole, core drilling was used (*n* = 145) and at three other boreholes, rotary drilling was applied (*n* = 76; 40 to 185 m b.g.l at each borehole). Therefore, sediment analysis was restricted to the samples obtained from core drilling.

### Carbon and sulphur analysis

To analyse the sediment geochemistry, samples were freeze-dried and ground to powder grain size. The carbon and sulphur contents were then determined using a combustion analyser (type: G4 Icarus, Bruker; detection limit, 0.01 wt.%, at Ruhr-University Bochum). In a O_2_ stream at a combustion temperature of 1250 °C, total carbon (C_tot_) was measured. Released CO_2_ was recorded by infrared spectrometric absorption measurement. The inorganic carbon content (C_inorg_) was determined during the reaction at 110 °C with perchloric acid (HClO_4_). With the difference between C_tot_ and C_inorg_, the C_org_ content was calculated. At combustion temperatures of 1400 °C and by infrared spectrometric determination of the released sulphur dioxide, total sulphur content was determined. The sulphide sulphur was also measured in a hot O_2_ stream and under increased pressure by combustion at 550 °C (Wisotzky 1994).

### Aqua regia digestion and dithionite solution

In order to obtain further information about the chemical composition of the sediment samples, they were dissolved with aqua regia and dithionite at the Hydrogeology Department, Ruhr-University Bochum. Aqua regia digestions serve to dissolve the solid sample matrix almost completely; silicate compounds remain as residues. A sample quantity of 200 mg was mixed with 50 ml of aqua regia (Suprapur) in Teflon tubes for the digestions. The microwave digestion device (MARS 6 One Touch, CEM) heats the samples to a temperature of 175 ± 5 °C. After cooling, the sample material was mixed with 20-ml distilled water in a volumetric flask. Insoluble filter residues were washed with diluted nitric acid (0.5 mol/l). The volumetric flask was then filled up to the mark with distilled water. Dithionite extraction will only dissolve amorphous and crystalline oxides and hydroxides from the samples. In the first step, 1 g of sample material was weighed into 50-ml centrifuge tubes, mixed with 40-ml Na citrate solution and then heated to 82 °C. Fifteen minutes before reaching the target temperature, 1 g Na dithionite was dissolved in 6 ml of a 1.25% Na hydroxide solution and added to the centrifuge tube. Subsequently, centrifugation was performed at 3000 rpm for 10 minutes. Finally, the liquid was poured from the sediment into a 250 ml volumetric flask with top filter. The collected extract was supplemented with distilled water up to the mark.

Using ICP-OES (type: Optima 8300, PerkinElmer, detection limit 0.1 mg/l), Fe and Mn concentrations were analysed in dithionite solutions and aqua regia digestions.

### Scanning electron microscope

The appearance and formation of Fe, Mn and Ca minerals was investigated and characterised using a scanning electron microscope (type: Zeiss/Leo Gemini 1530, resolution 5 nm, at Ruhr-University Bochum) on 10 samples from the sediments of the drilled cores (1 sample from the Quaternary, 9 samples from the Tertiary aquifer). In addition, spectral analysis (wavelength-dispersive point measurements) was used to obtain chemical information of individual components.

### Water analysis and hydrogeochemical modelling

For the evaluation of groundwater hydrochemistry, the local waterworks provided water analyses of four shallow wells (period 1989 to 2017, *n* = 319), four deep wells (1991 to 2017, *n* = 311) and one multilevel well (2006 to 2018, *n* = 188) (laboratory of the local water supplier, ICS 1100, Thermo Fisher Scientific, ions passed all QA requirements for efficiency, retention time, asymmetry and resolution). To process the data, analyses carried out several times a year were averaged and the individual parameters plotted against time in order to evaluate the annual changes in water chemistry. The presentation of each individual well would have been far too extensive, so the individual parameters were averaged for shallow and deep wells. Hydrogeochemical and physico-chemical parameters from the multilevel well were compared between the years 2006, 2009, 2014 and 2018. Water analyses until 2006 were published in Mäurer and Wisotzky (2007) and are presented here for comparison.

In order to derive statements on possible dissolution and precipitation reactions, a hydrogeochemical modelling of mineral saturation indices was carried out using the software PhreeqC 3.4 (Parkhurst and Appelo 2013).

### Mixing ratio and mixture modelling

Previous investigations (Mäurer and Wisotzky 2007) have determined a groundwater flow from the Quaternary sediments to the underlying Tertiary deposits resulting from groundwater extraction from deep wells. Consequently, the current proportion of admixture is calculated according to the following Eq. 3:

3 $$ \mathrm{X}\ \left[\%\right]=\frac{c_{\mathrm{uGwLt}1}-{c}_{\mathrm{uGwLt}0}}{c_{\mathrm{oGwLt}1}-{c}_{\mathrm{uGwLt}0}}\ast 100 $$

where *X* [%] is the ratio of Quaternary groundwater in the sample,

*c*_uGwLt0_: is the Cl^-^ concentration of groundwater in Tertiary sediments at time *t*_0_,
*c*_uGwLt1_: is the Cl^-^ concentration of groundwater in Tertiary sediments at time *t*_1_,
*c*_oGwLt1_: is the Cl^-^ concentration of groundwater in Quaternary sediments at time *t*_1_.

In a further step, mixing modelling was carried out on the basis of the determined proportion of admixture in order to simulate the changed conveying conditions. Since the shallower and deeper waters are in mutual contact in the transition zone from Quaternary to Tertiary aquifer, water analyses from the deep Quaternary and from the upper Tertiary sediments were used for modelling.

## Results

### Carbon and sulphur analysis

**Fig. 1:**
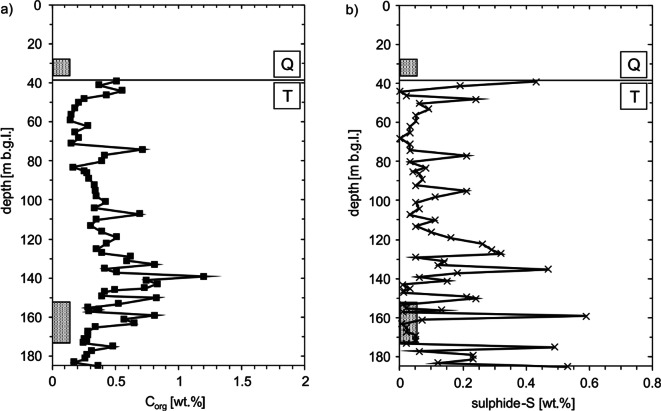
Measured distribution of *C*_org_ (**a**) and sulphide sulphur (**b**) contents in the core sediments. The Quaternary-Tertiary boundary is abbreviated with Q and T. The boxes at approx. 30 m b.g.l. mark the screening of the shallow well and the screening of the deep well at approx. 160 m b.g.l.

The *C*_org_ content of the Tertiary sediments varies between 0.15 and 1.20 wt.% (Fig. 1a). The maximum values were measured in individual peaks at a depth of approx. 140 m. The mean *C*_org_ content is 0.42 wt.%.

The sulphide sulphur contents show a substantially higher fluctuation (Fig. 1b). Minimum contents are below the detection limit; the maximum content is 0.59 wt.% at a depth of 159 m. All in all, an average sulphide sulphur content of 0.12 wt.% could be determined.

### Aqua regia digestion and dithionite solution

**Fig. 2:**
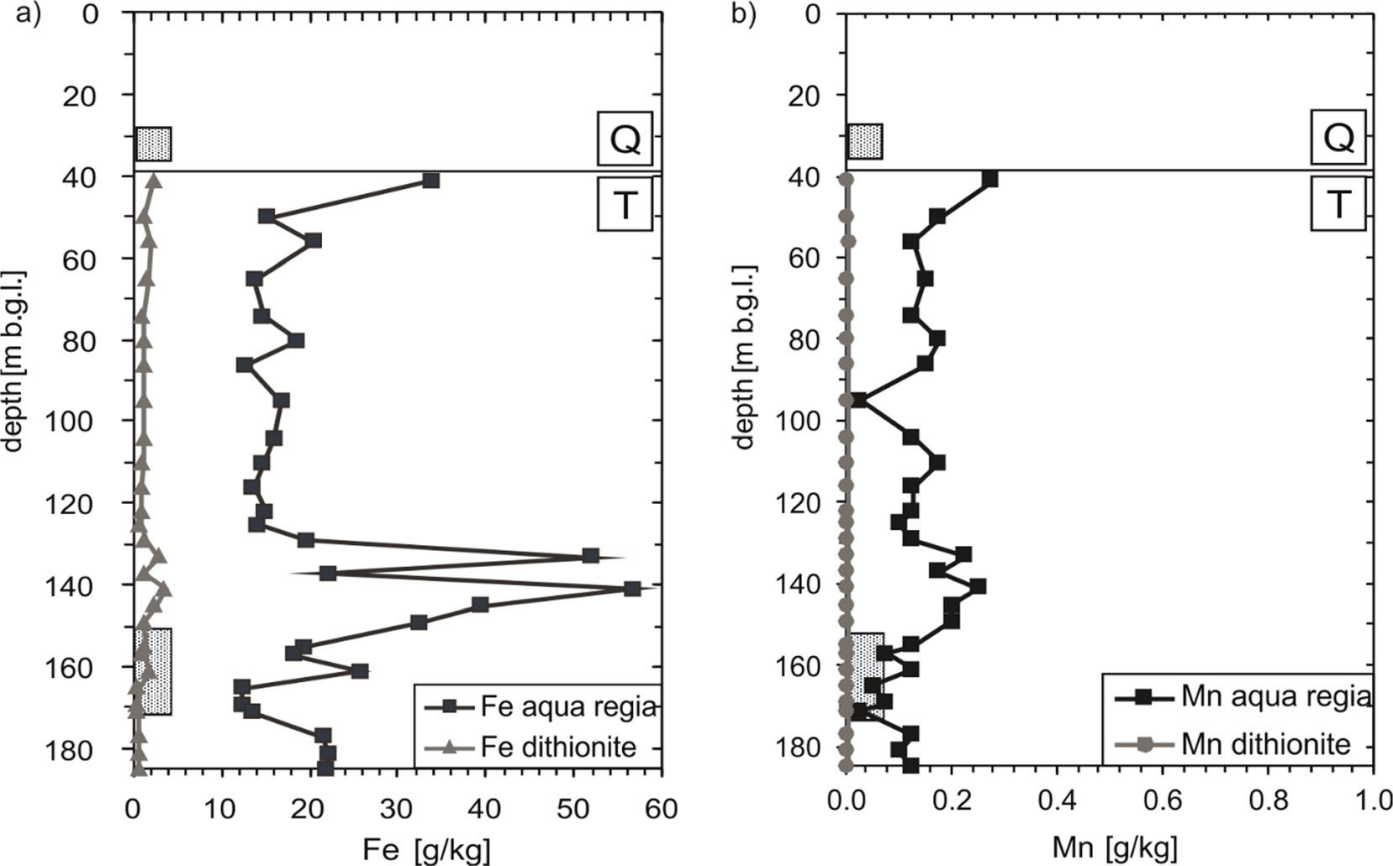
Measured distribution of Fe (**a**) and Mn (**b**) in the core sediments; dissolved by aqua regia in black, dissolved by dithionite in grey. The Quaternary-Tertiary boundary is abbreviated with Q and T. The boxes at approx. 30 m b.g.l. mark the screening of the shallow well and the screening of the deep well at approx. 160 m b.g.l.

The mean Fe content dissolved by Aqua regia is 21.7 g/kg (Fig. 2a). The values show a more or less constant behaviour in the upper about 90 m of the Tertiary sediments, slightly below the average Fe content. In the depth range between 130 and 150 m b.g.l., much higher contents are observed. Over the entire depth, the contents vary between 12.3 and 56.5 g/kg. The dithionite-soluble Fe shows substantially smaller quantities but generally resembles the depth development of total Fe contents. On average, the dithionite-soluble Fe is 1.26 g/kg, with values varying between 0.43 and 3.45 g/kg.

The Mn content of the Tertiary sediments is substantially lower compared to the Fe content (Fig. 2b). Manganese dissolved by aqua regia fluctuates between 0.03 and 0.28 g/kg over the entire depth. The average content is 0.14 g/kg. With the exception of the sample at a depth of 56 m b.g.l. (0.005 g/kg), the dithionite-soluble Mn lies below the analytical detection limit. Total Fe and Mn contents are very well comparable to values reported by Banning and Rüde (2010) for marine Tertiary sediments in the Lower Rhine Embayment.

### Scanning electron microscope

**Fig. 3:**
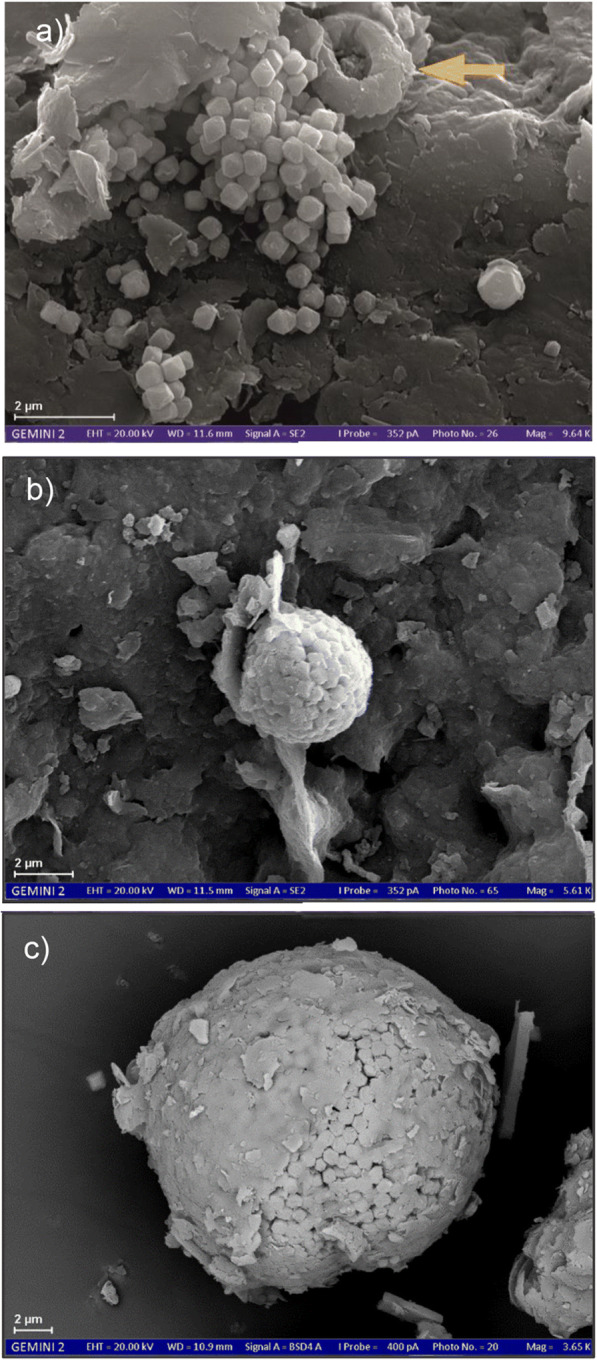
Scanning electron microscope image: subcubic pyrite, 50 m b.g.l., yellow marker, Ca carbonate component (**a**); framboidal pyrite, 141 m b.g.l (**b**); framboidal pyrite partially coated with clay particles, 171 m b.g.l (**c**)

The Quaternary sediment at a depth of 25 m b.g.l. consists mainly of quartz grains and clays, whereby the clay particles are often deposited on the quartz grains. Pyrite and manganese compounds are not found in the Quaternary aquifer. This changes substantially in the Tertiary aquifer. Pyrites are often found down to a depth of 141 m b.g.l. where they mainly exhibit subhedral, i.e. nearly cubical, shapes (Fig. 3a). At greater depths (below 141 m b.g.l.), the pyrite is mainly framboidal (Fig. 3b). Pyrite minerals, especially the framboidal forms, are frequently covered with clay particles (Fig. 3c). Several spectral analyses indicate the presence of Fe compounds and especially Fe carbonates in the Tertiary sediments. However, these do not have a distinct crystal structure. Round Ca carbonate components (Fig. 3a, top central) were also found throughout the Tertiary aquifer and suggest the presence of marine microfossils such as coccoliths. The occurrence of pyrite, siderite and calcite for marine Tertiary sediments in the region was also observed by Banning and Rüde (2010).

### Water analysis and hydrogeochemical modelling

#### Shallow wells

**Fig. 4:**
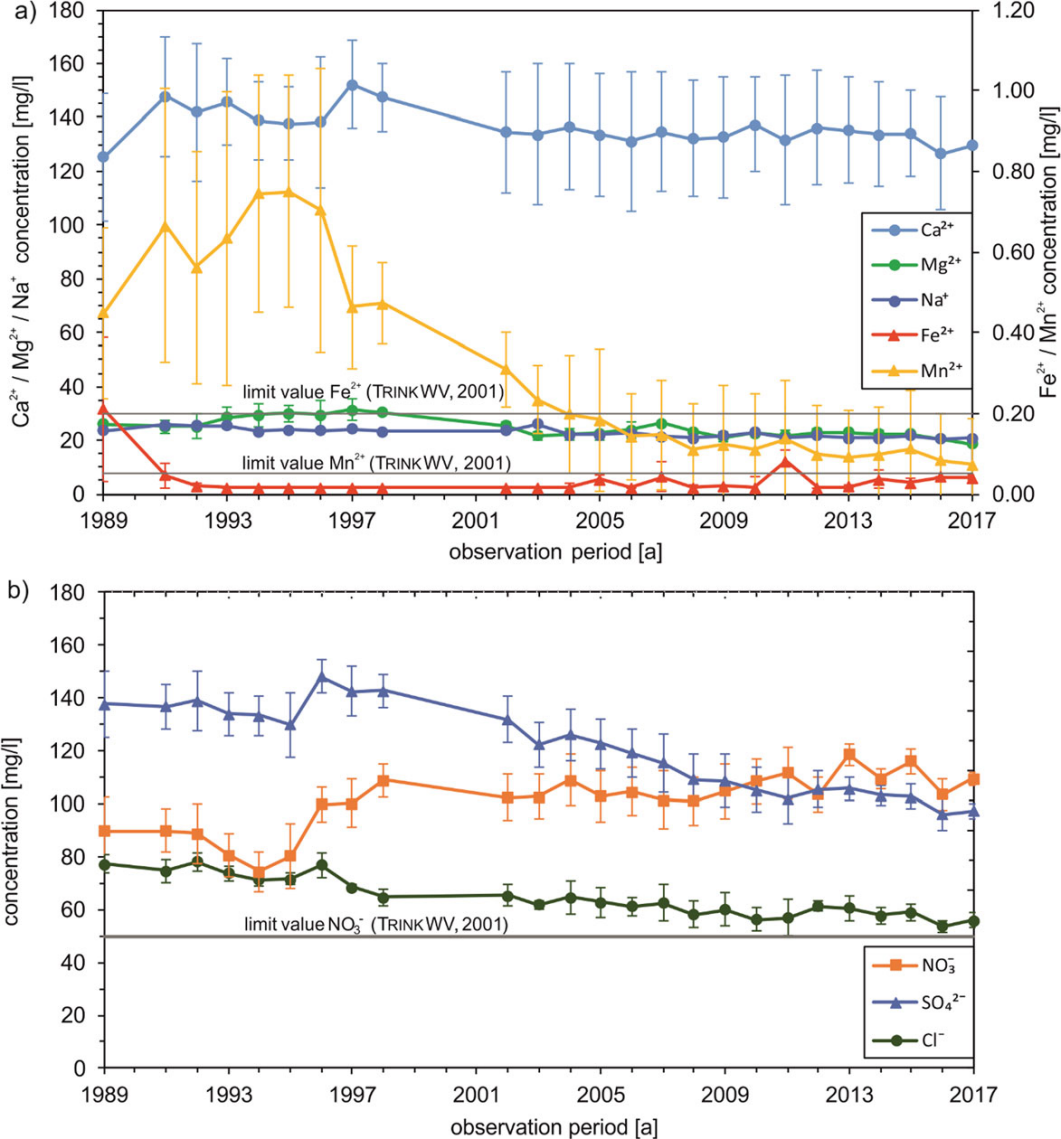
Hydrogeochemical development of shallow wells: cations (**a**), anions (**b**); whiskers indicate standard deviations

The four shallow wells filtered in the Quaternary aquifer show a slightly decreasing trend in Ca^2+^ concentrations since the late 1990s (Fig. 4a). After a brief increase in concentration in the first few years of observation with a maximum value of 152 mg/l, there was a decrease to about 124 mg/l by 2017. The Mg^2+^ and Na^+^ concentrations are almost constant. Manganese concentration shows a clear decrease to 0.08 mg/l by 2017, after the concentration initially rises to 0.75 mg/l (1995). Despite a decreasing trend, the Mn^2+^ concentration is above the drinking water threshold value of 0.05 mg/l over the entire observation period (Trinkw 2001). The Fe^2+^ concentration decreases in the first years from 1989 (0.21 mg/l) to 1993 (< 0.02 mg/l). Only slight fluctuations can be observed in the following years. At the end of the observation period, a slight increase to 0.05 mg/l has been observed. Thus, the drinking water limit of 0.2 mg/l (Trinkw 2001) is exceeded only minimally in the first year of observation.

The average NO_3_^-^ concentration in the shallow wells (Fig. 4b) is permanently above the limit value of the drinking water regulation of 50 mg/l (Trinkw 2001). In addition, the concentration increased from 87.8 (1989) to around 109 mg/l (2017). Conversely, the SO_4_^2-^ concentration shows decreasing values with occasional fluctuations. The average concentration was 138 mg/l in 1989 and decreased to about 97.3 mg/l by 2017. Similarly, the Cl^-^ concentration in the shallow wells shows a decreasing trend. In 1989, the initial concentration was 77.0 mg/l and it decreased to 55.7 mg/l by 2017.

The pH value is circumneutral and no fluctuations can be observed over the entire observation period (data not shown).

**Fig. 5:**
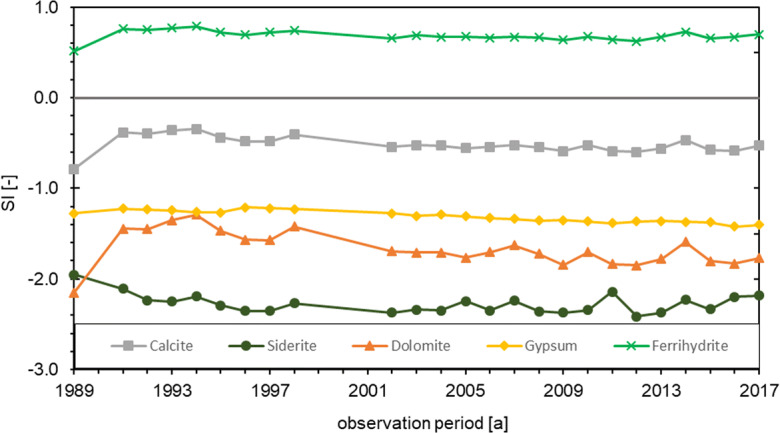
Development of mineral saturation indices in groundwater from the shallow wells

The saturation indices of calcite, siderite, dolomite and gypsum show negative values over the entire period and thus undersaturation (Fig. 5). Calcite SI is the highest with approx. −0.5 and siderite SI the lowest with approx. −2.2. All saturation indices show only slight fluctuations. The saturation index of ferrihydrite is permanently in the positive range and is therefore the only studied mineral which is supersaturated. Nevertheless, no Fe hydroxide precipitations and also no reduction of the yield of the shallow wells were observed. The CO_2_ partial pressure (average 3.33 vol.%) shows some fluctuations over the years. However, there are no clear or steady increases to be seen.

#### Deep wells

**Fig. 6:**
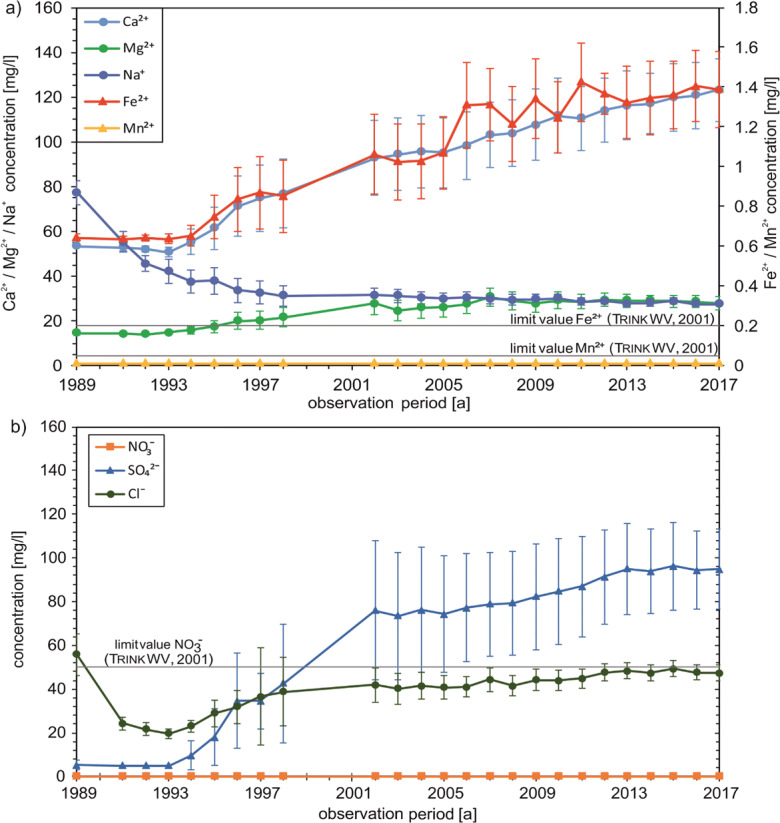
Hydrogeochemical development of deep wells: cations (**a**), anions (**b**); whiskers indicate standard deviations

In the deep wells, some concentration differences can be observed in comparison to the shallow wells. The Ca^2+^ concentration shows a clear and steady increase since 1994 (Fig. 6a). In the beginning, the concentration was almost constant at approx. 50 mg/l, before continuously rising to 123 mg/l by 2017. The Mg^2+^ concentration also points out a slight increase but remains constant from 2007 onwards. Another trend is shown by the Na^+^ concentration, which clearly decreases from 77.3 mg/l at the beginning of the observation period to 31.2 mg/l in 1998 and then only slightly to 27.6 mg/l by 2017. From 1994 onwards, an increasing trend of the average Fe concentration in the deep wells of water catchment can be observed. The initial concentration in 1989 was around 0.64 mg/l and rises to 1.39 mg/l by 2017. Consequently, the limit value of the drinking water regulation is permanently exceeded. The mean Mn^2+^ concentration shows no changes and is permanently below the detection limit of 0.02 mg/l.

In contrast to shallow wells, the NO_3_^-^ concentration in deep wells is below the analytical detection limit of 1 mg/l in all observation years (Fig. 6b). In addition, no O_2_ could be detected (values constantly below 0.1 mg/l, data not shown). The SO_4_^2-^ concentration, on the other hand, increases substantially from 1994 onwards, as does the Ca^2+^ and Fe^2+^ concentration, from 5.38 to 94.9 mg/l. The mean Cl^-^ concentration first shows a decrease to 19.7 mg/l but from 1994 an increase to 47.7 mg/l over the years.

Even in the deep wells, pH is circumneutral, but a slight decrease from 7.5 to 7.3 can be observed (data not shown).

**Fig. 7:**
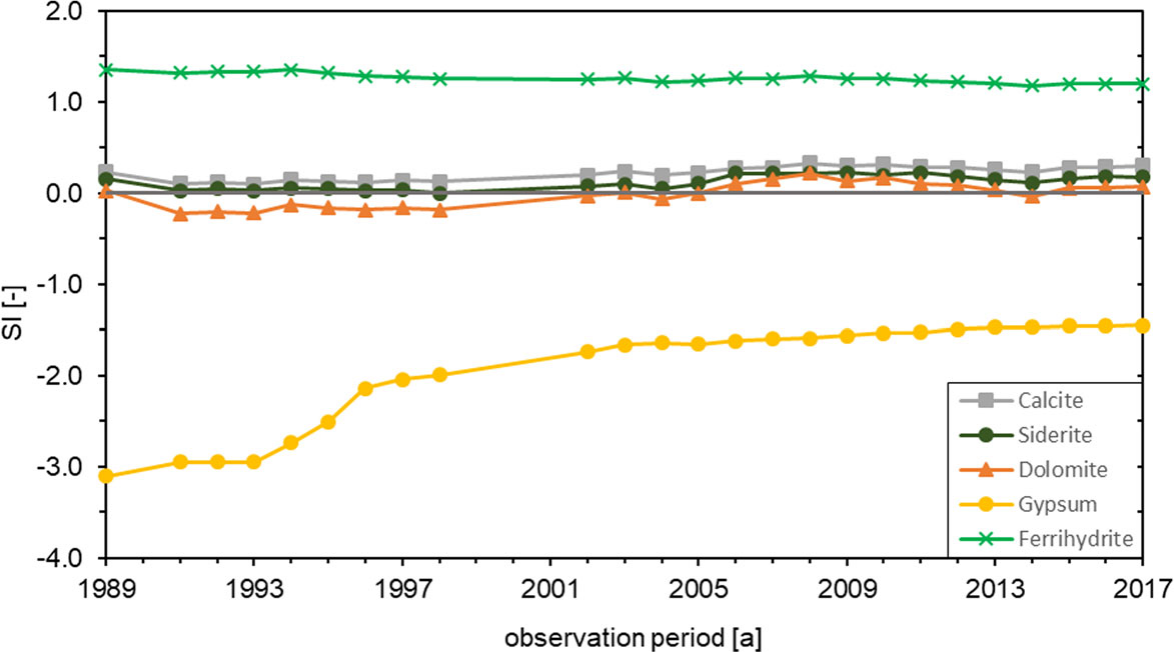
Development of mineral saturation indices in groundwater from the deep wells

The hydrogeochemical modelling of the deep wells also shows different results than in the shallow wells (Fig. 7). A slight supersaturation of the minerals calcite and siderite can be observed. In addition, the saturation indices of calcite, siderite and dolomite rise from 1998 onwards; dolomite is slightly supersaturated from 2005. Gypsum is clearly undersaturated even in the deep wells, although its SI rises with increasing SO_4_^2-^ concentrations from 1993. The saturation index of ferrihydrite is permanently > 1 and thus shows slightly more positive values than in the shallow wells. Nevertheless, no precipitation and no reduction in the yield of the deep wells were observed here either. The CO_2_ partial pressure rises steadily (0.89 up to 1.55 vol.%), similar to the Ca^2+^, Fe^2+^ and SO_4_^2-^ concentration, but reacts with a delay of 1 year, so the total increase of 0.66 vol.% can only be observed from 1995 onwards.

By hydrogeochemical equilibrium modelling, a statement can be made as to which quantities of selected minerals (calcite, siderite and ferrihydrite) are dissolved or precipitated until equilibrium is reached. For this purpose, averaged water analyses from 2017 are used. It is assumed that the measured total iron (21.66 g/kg) minus sulphide iron (1.05 g/kg) and minus ferrihydrite (1.26 g/kg, from dithionite-soluble Fe^2+^) is present as siderite. Thus, 19.4 g/kg (= 0.2 wt.% C_inorg_) of the Fe^2+^ content is probably present as siderite. Consequently, the remaining 0.27 wt.% C_inorg_ is assumed to be calcite. This results in an average calcite content of 22.5 g/kg in the Tertiary marine sands. Equilibrium modelling shows an average precipitation of 0.36 × 10^-4^ mmol/l calcite until equilibrium is reached. In addition, 9.31 × 10^-6^ mmol/l siderite and 6.34 × 10^-7^ mmol/l ferrihydrite should precipitate to equilibrium.

#### Multilevel well

**Fig. 8:**
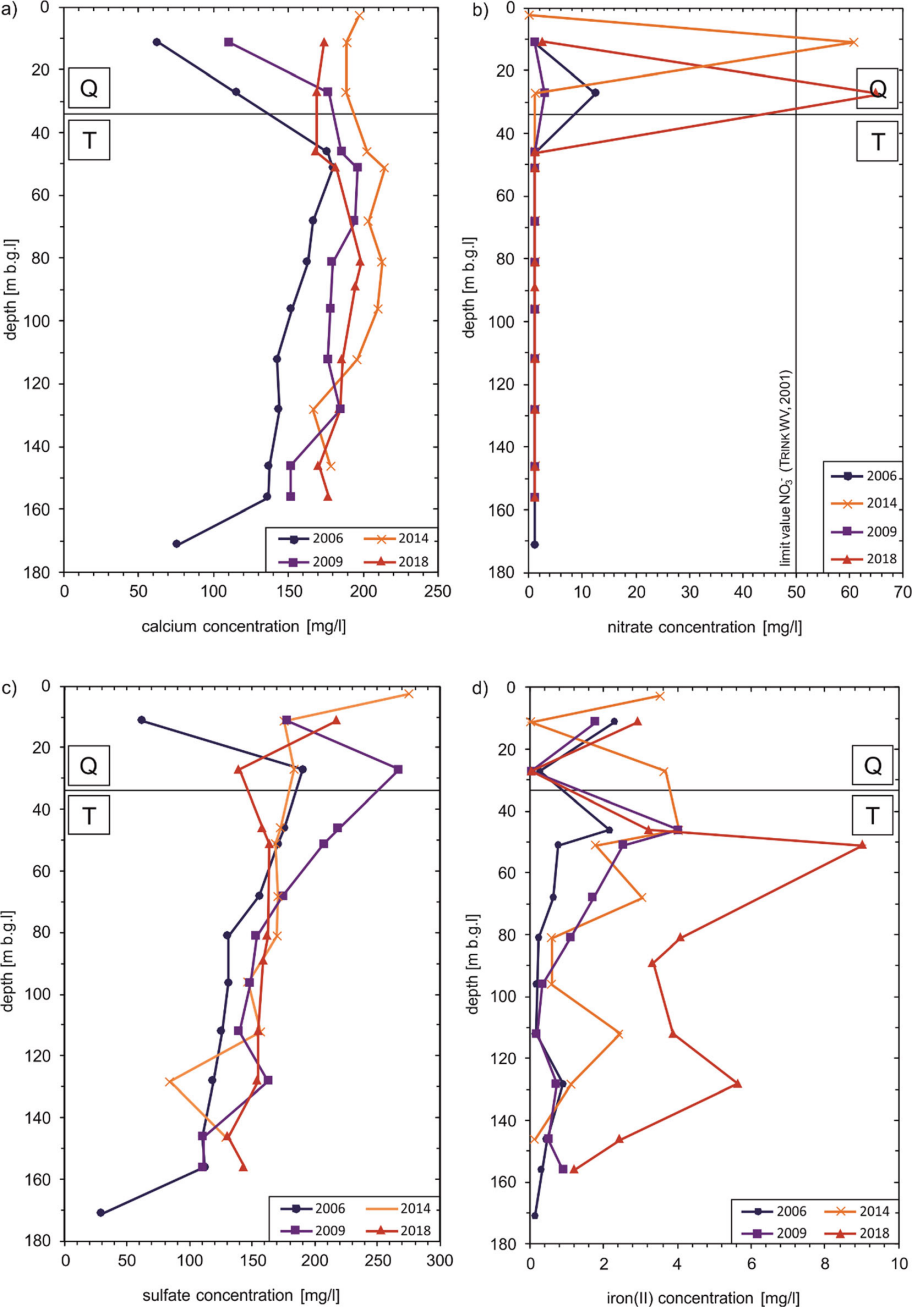
Hydrogeochemical development (selected ions) of groundwater from the multilevel well: calcium (**a**), nitrate (**b**), sulphate (**c**) and iron (**d**)

In the multilevel well, the same parameters show peculiarities as in the deep wells. For example, the Ca^2+^ concentration reaches maximum values below the Quaternary-Tertiary boundary in order to drop slightly to the final depth (Fig. 8a). A comparison between 2006 and 2018 shows that the Ca^2+^ concentration rises slightly over nearly the entire depth. Bicarbonate shows no substantial temporal changes. However, the concentration also rises slightly at the Quaternary-Tertiary boundary and is constant again in the following metres (data not shown). Nitrate is present in the Quaternary aquifer and continues to rise over the years. Immediately after the transition to the Tertiary aquifer, the NO_3_^-^ concentration falls below the detection limit in all years (Fig. 8b). The SO_4_^2-^ concentration rises in 2006 and 2009 initially still in the lowest metres of the Quaternary aquifer and decreases slowly over the further depth (Fig. 8c). In 2014 and 2018, on the other hand, the SO_4_^2-^ concentration initially drops in the lowest metres of the Quaternary aquifer but then rises slightly after the transition to the Tertiary aquifer and then also drops over the further depth. In line with this, an increase can also be observed in the Fe^2+^ concentration shortly after the Quaternary-Tertiary limit. In 2018, the Fe^2+^ concentration rises at a depth of 50 m b.g.l. to a maximum concentration of 9.01 mg/l (Fig. 8d). The pH value is circumneutral and shows a very small decrease between 2006 and 2018. Redox potential drops similarly to some ion concentrations at the Quaternary-Tertiary boundary and indicates reducing conditions (data not shown).

**Fig. 9:**
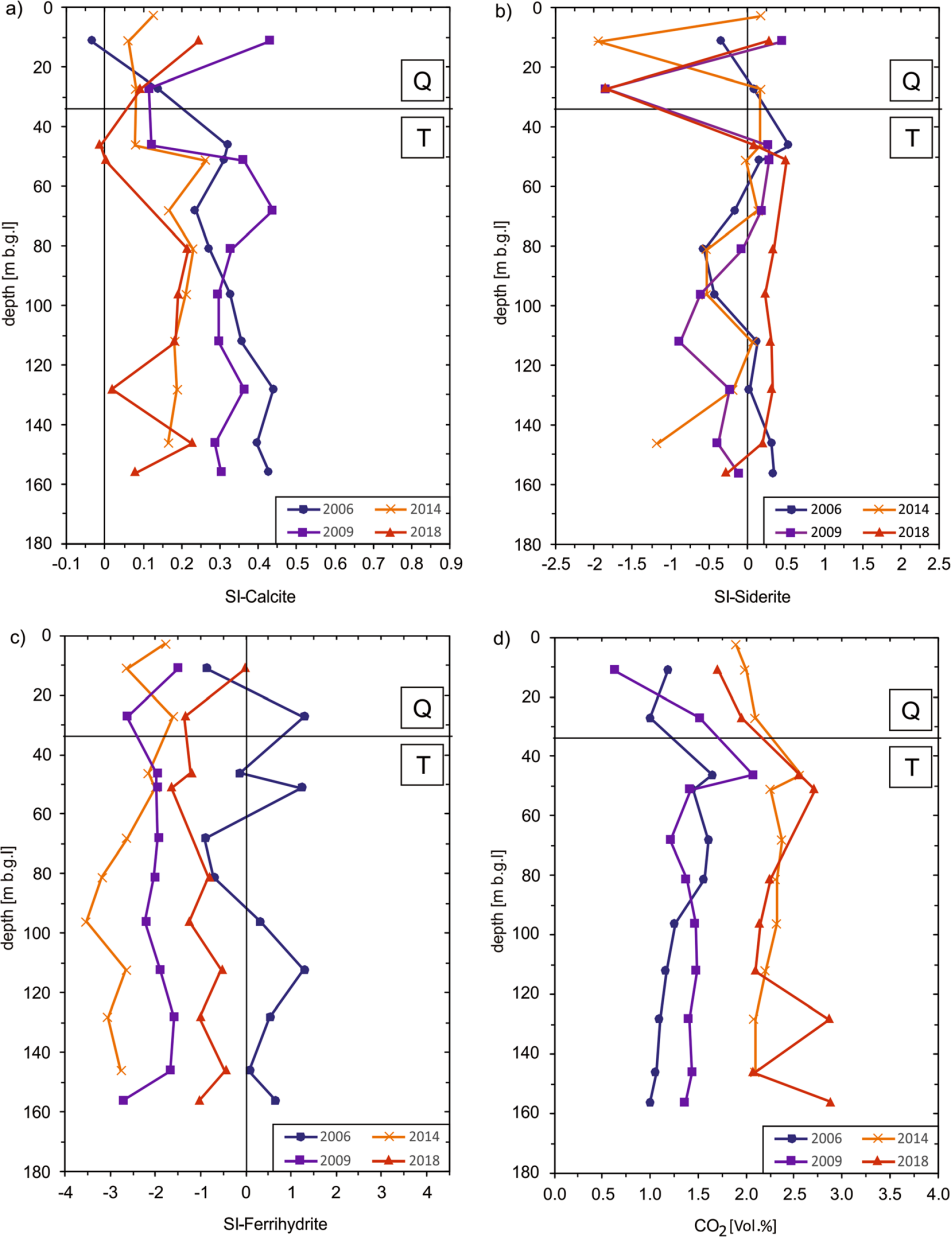
Development of the saturation indices of calcite, siderite and ferrihydrite as well as the CO_2_ partial pressure of groundwater from the multilevel well

Similar to the concentrations described above, the saturation indices also show changes, especially at the Quaternary-Tertiary boundary. Calcite is slightly oversaturated over the entire depth. In a range of 25 to 45 m b.g.l., the supersaturation is substantially lower. In 2018, the saturation index even falls slightly into the negative range. Overall, it can be observed that the slight oversaturation of calcite is lower compared to the years before (Fig. 9a). Siderite saturation also rises substantially shortly after the transition to the Tertiary aquifer and shows only slight fluctuations over the remaining depth (Fig. 9b). The saturation index of ferrihydrite, on the other hand, does not show a substantial increase at the Quaternary-Tertiary border, but a few measurement levels indicate supersaturation in 2006. In the following years, ferrihydrite is partly clearly undersaturated over the entire depth (Fig. 9c). The CO_2_ partial pressure, on the other hand, increases again in the upper meters of the Tertiary aquifer in all years (Fig. 9d). Dolomite is permanently undersaturated and does not exhibit any special features.

### Admixture ratio and mixture modelling

**Fig. 10:**
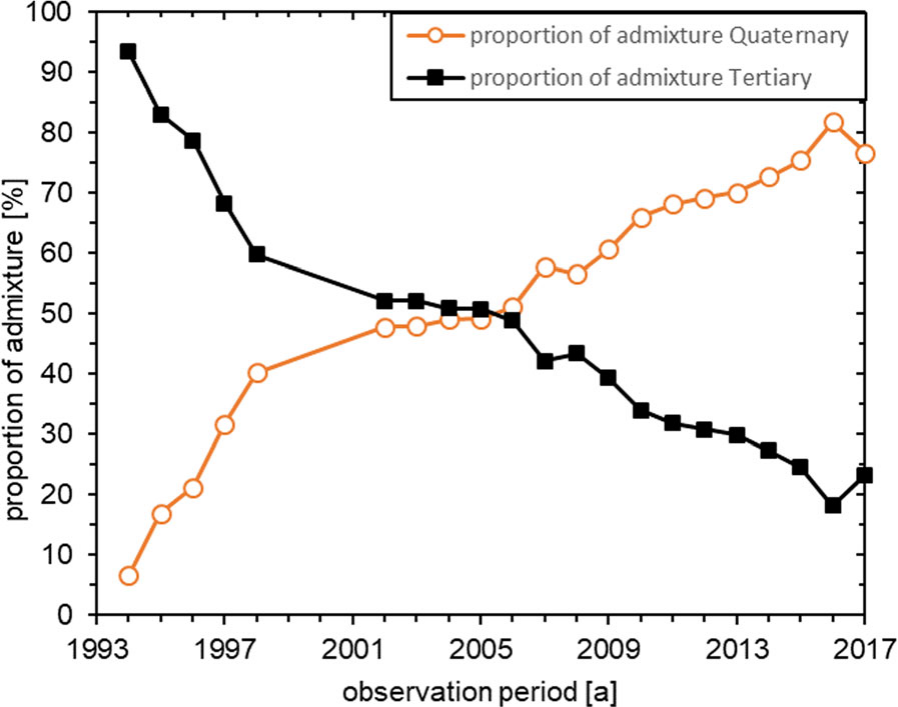
Mean mixing proportion of average Quaternary and Tertiary groundwater end members

The average proportion of admixture from the Quaternary groundwater horizon was just under 10% in 1994 and rose to around 50% by 2002. From 2002 to 2006, an almost constant proportion of admixture was determined. In the following years, the proportion of admixture from the Quaternary aquifer rose to approx. 75% in 2017 (Fig. 10).

The mixture modelling (Table 1) shows the mixed waters with a calcite saturation index of −0.01 which is almost in equilibrium. Dolomite is undersaturated with −0.66 and ferrihydrite is clearly undersaturated with a saturation index of −3.44. The saturation index of siderite is the only one in the positive range with 0.98 and thus indicates supersaturation.

**Table 1:** Mixture modelling: saturation indices of groundwater end members (Quaternary and Tertiary aquifers, sample 2018) and of mixed waters

	Groundwater Quaternary	Groundwater Tertiary	Mixed water
SI Calcite [-]	−0.21	0.48	−0.01
SI dolomite [-]	−1.04	0.21	−0.66
SI siderite [-]	−1.28	0.86	0.98
SI ferrihydrite [-]	−3.65	−0.53	−3.44

## Discussion

The results of carbon measurements in the Tertiary aquifer show relatively high C_org_. Compared to values from previous investigations with an average of 0.22 wt.% (Mäurer and Wisotzky 2007), C_org_ is substantially higher in the sediments studied here (0.42 wt.%). This deviation can probably be attributed to the C_org_-containing drilling fluid used. Nevertheless, about 0.22 wt.% of C_org_ is assumed to be present in the Tertiary aquifer, whereby probably not all of it is available as a reducing agent for chemoorganotrophic NO_3_^-^ reduction due to limited reactivity (Hartog et al. 2004). The average sulphide sulphur content of 0.12 wt.% is more consistent with the investigations of Mäurer and Wisotzky (2007). They found a slightly lower content of 0.09 wt.% in the Tertiary marine sands, while Banning and Rüde (2010) detected an average of 0.33 wt.% S_pyrite_ (converted from Rietveld-quantified XRD results). With 0.12 wt.% sulphide sulphur, a relatively large amount of reducing agent is available for chemolithotrophic NO_3_^-^ reduction. This value is readily comparable with data from Hartog et al. (2005) reporting an average of 0.14 wt.% S for late Tertiary sediments from the nearby eastern Netherlands.

Scanning electron microscope analysis also confirms a high content of sulphide sulphur in the Tertiary aquifer, as much pyrite could be detected in both cubic and framboidal forms. However, pyrites coated with clay particles were found repeatedly. Thus, a part of the pyrite may not be available for NO_3_^-^ reduction, since the reactive surface does not get into reaction contact with the groundwater. According to Jorgensen et al. (2009), only 50% of sulphide sulphur is reactive in most cases.

From the results of the aqua regia digestions and the dithionite solutions, the composition of the sedimentary Fe pool (excluding silicate iron compounds) can be estimated. An average proportion of 5.82% of Fe is present as amorphous and crystalline Fe oxides and hydroxides in the Tertiary aquifer. The mean sulphide sulphur content of 0.12 wt.% corresponds to a Fe sulphide content of 1.05 g/kg, which accounts for 4.85% of the total Fe content. Thus, there is probably a remaining 19.4 g/kg present mainly as Fe carbonate in the Tertiary marine sands.

The hydrogeochemical analyses reflect some properties of the previously described sediments the waters are in contact with. In the water from the Quaternary aquifer (shallow wells), analysed ion concentrations show only slight fluctuations over the observation period. The slow decrease in Cl^-^ and SO_4_^2-^ concentrations observed over the years may be due to lower fertiliser input. The NO_3_^-^ concentration, on the other hand, increases over the entire observation period. In recent years, a slight decreasing trend has also been observed. The Mn^2+^ concentration decreases substantially due to the occurrence of O_2_ in the aquifer since 1998, as Mn^2+^ ions are oxidised and precipitated from the groundwater (Nolte and Bergmann 2007), probably as MnO_2_. The Quaternary waters show high CO_2_ partial pressures compared to atmospheric values (0.038 vol.%). However, since no increasing trend can be observed, no conclusions can be drawn about a reaction like organotrophic NO_3_^-^ reduction. If these waters are modelled in contact with atmospheric CO_2_ partial pressure and CO_2_ is outgassed, calcite is supersaturated with an equilibrium saturation index of 1.2. Since no sintering by calcite is observed in the shallow wells, the waters are probably only in contact with the atmosphere for a short time on their way to water treatment and no precipitation occurs. With a longer lasting production stop in the shallow wells, precipitation could occur. Similar observations are made for ferrihydrite (amorphous Fe hydroxide). A weak supersaturation is present over the entire observation period. A precipitation of ferrihydrite would therefore be possible, but precipitations are not observed here either.

In contrast to the shallow wells, the deep wells have seen substantial increases in Ca^2+^, SO_4_^2-^ and Fe^2+^ concentrations since 1994. Reduction of NO_3_^-^ and related subsequent reactions are decisive for the increase of these concentrations in the Tertiary groundwater. By pumping groundwater from the deep wells and lowering groundwater levels, Quaternary water with a high NO_3_^-^ concentration is transported into the Tertiary aquifer. The admixture proportion of Quaternary groundwater was determined to be 75%. Thus, after an almost constant period up to 2006, it increases by 25%. Despite the addition of Quaternary groundwater and consequently a high NO_3_^-^ input, the NO_3_^-^ concentrations over the entire observation period in the Tertiary aquifer are below the analytical detection limit. The increase of SO_4_^2-^ and Fe^2+^ concentrations, with a simultaneous absence of NO_3_^-^ in the reduced Tertiary marine sands, indicates a chemolithotrophic denitrification. This reaction leads to a release of protons which would have to lower the pH value. However, since there are no changes in the pH value and the concentrations of Ca^2+^ and Mg^2+^ as well as the CO_2_ partial pressure also increase, the released H^+^ ions are buffered by dissolving carbonates. From the increase in Ca^2+^ concentration between 1989 and 2017, a dissolved calcite quantity of 0.17 g/l was calculated, which corresponds to 0.76% of the total calcite content in the sediment.

In addition to the increase in Ca^2+^ concentration, the saturation indices of carbonates also increase, since carbonates are dissolved by the release of protons. Consequently, there is no precipitation of calcite, as was previously determined in equilibrium modelling. A release of Ca^2+^ and HCO_3_^-^ ions occurs; consequently, the waters are increasingly supersaturated with calcite. The buffering of the protons therefore triggers a solution of the carbonates, although the saturation index of calcite increases. Furthermore, no further siderite precipitations are to be expected, since the formation of siderite is usually kinetically inhibited (Jensen et al. 2002). The modelling results for ferrihydrite show saturation indices which always lie in the oversaturated range. A precipitation of this mineral would therefore be possible and would lead to incrustations in the form of ochcification. However, since no precipitation of ferrihydrite in the deep wells has been observed so far, the time between extraction and water treatment may be too short for ochcification to occur. If production is stopped for a longer period, precipitation could occur.

The results of the multilevel well underline the conclusion of the chemolithotrophic NO_3_^-^ reduction and the associated follow-up reactions. In addition, a change in the concentrations and in the saturation indices at the Quaternary-Tertiary boundary is evident. If the nitrate-containing water from the Quaternary aquifer comes into contact with the pyrite-containing sediment, the described reactions occur directly in the upper metres (at a depth of approx. 50 m) of the Tertiary aquifer. A suchlike highly reactive redox transition zone was also described in Cretaceous sediments by Banning et al. (2013b) and for Tertiary sediments by Banning and Rüde (2010).

Results of mixture modelling also do not suggest considerable mineral precipitation, as the saturation indices of all studied mineral phases are negative. Only siderite is supersaturated (SI = 0.98). Since siderite precipitation is often a kinetically inhibited process, it is not necessarily to be expected despite the supersaturation. It has, however, been observed in the redox transition zone between Tertiary and Quaternary sediments, leading to a content of 8.51 wt.% siderite in the sediments (Banning and Rüde 2010).

In the future, it remains to be seen whether the steady solution of pyrite and the associated release of Fe^2+^, SO_4_^2-^ and indirectly Ca^2+^ and HCO_3_^-^ ions will lead to the supersaturation of some minerals. Above all, the Fe^2+^ concentration and thus the saturation states of Fe minerals must be closely observed.

Due to the high sulphide S content of 0.12 wt.%, a vertical nitrate breakthrough time in the deep wells has been calculated according to DWA (2015). In contrast to DWA (2015), the shift of nitrate from the Quaternary sediments to the filter screen in the Tertiary layers is considered here. This results in a vertical flow distance of 124 m from the Quaternary-Tertiary boundary to the top of the filter screen section. Nitrate concentration of the Quaternary aquifer was adopted from the studied time series and is 110 mg/l in 2017. Groundwater recharge of 150 l/a is assumed for the calculation, representing a typical value for the Lower Rhine valley (Bogena et al. 2005). Based on these data, a vertical nitrate breakthrough time to the deep well filter screens of about 13,000 years was calculated. Since stoichiometrically, 4 moles of protons are released per mol pyrite during pyrite oxidation and a vertical breakthrough time of the protons after consumption of the sediment carbonate buffer capacity (calcite and siderite) of about 1,500 years was determined. This number includes about 900 years of buffering by calcite dissolution and about 600 years by siderite dissolution whereby the latter is considered a maximum value: siderite dissolution is limited under neutral conditions and presence of oxidising agents due to surface passivation by ferrihydrite precipitation (Duckworth and Martin 2004; Seibert et al. 2016).

Overall, it becomes clear that for water suppliers, NO_3_^-^ is not only a direct problem if threshold values of drinking water regulations are exceeded. Tertiary and Quaternary sediments in the area studied here form one hydraulically connected aquifer, with virtually nitrate-free reduced deep water which is used to dilute shallow groundwater with elevated nitrate concentrations. However, NO_3_^-^ reduction induced by pulling down shallow oxic groundwater to deeper layers during pumping can trigger a number of subsequent reactions potentially leading to deteriorating raw water quality and sintering or ochcification with associated specific well yield reduction. In addition, mobilisation of unwanted trace elements like Ni, U, As, etc. from aquifer sediments is possible through NO_3_^-^ input (Houben et al. 2017; Banning et al. 2013a; Abraitis et al. 2004), but this has not yet been observed in the studied aquifer.

## Conclusions

In shallow Quaternary groundwater, NO_3_^-^ concentrations of > 100 mg/l have been determined over many years and the proportion of Quaternary groundwater in the deep wells now amounts to about 75 %. This results in massive NO_3_^-^ input into the Tertiary groundwater. Despite these high NO_3_^-^ inputs, concentrations in deep groundwater are still below the analytical detection limit. Therefore, a NO_3_^-^ reduction must occur, which is also confirmed by changed hydrochemistry. Since 1994, Fe^2+^ and SO_4_^2-^ concentrations have been steadily increasing, indicating a chemolithotrophic NO_3_^-^ reduction. Sediment analyses showed an average sulphide sulphur content of 0.12 wt.%. Consequently, there is substantial potential for chemolithotrophic NO_3_^-^ reduction — a vertical nitrate breakthrough time to the deep well filter screen of about 13,000 years was calculated. Protons released during this reaction are buffered by carbonate dissolution, which consequently also substantially increases Ca^2+^ concentrations and CO_2_ partial pressures. Of the total calcite contained in the sediment, < 1% has been dissolved so far and a vertical breakthrough time of the protons after consumption of the carbonate buffer capacity of about 1,500 years was determined.

The release of Ca^2+^ ions also increases the saturation indices of carbonates. Nevertheless, due to the necessary buffering, carbonate precipitation will probably not take place but rather further dissolution. Modelled mixing of Quaternary and Tertiary groundwater end members also does not suggest considerable mineral precipitation; only siderite precipitation seems to be a realistic actual scenario. However, once NO_3_^-^ reduction ceases due to complete consumption of the natural reduction capacity in sediments, a NO_3_^-^ breakthrough to extraction wells might well be accompanied by Fe hydroxide precipitation and the risk of well clogging.

Thus, the entire hydrochemistry is strongly influenced by NO_3_^-^ input and above all by NO_3_^-^ reduction products and secondary reactions. In addition, a further progressive change and mineral precipitations are possible in the future. These should be taken into account for future groundwater management, as well as the fact that despite the calculated long-term stability in the area studied here, the geogenic NO_3_^-^ reduction capacity in used aquifer systems is a limited resource.

This study underlines the importance of thorough temporal, spatial and depth-dependent hydrogeochemical monitoring and assessment in order to identify and characterise water quality-related challenges and develop counterstrategies in time, especially in view of increased water demands to be expected under ongoing climate change conditions.

## Data Availability

PhreeqC, publicly available code for hydrogeochemical calculations: https://www.usgs.gov/software/phreeqc-version-3
